# Neural Network Mechanisms Underlying Stimulus Driven Variability Reduction

**DOI:** 10.1371/journal.pcbi.1002395

**Published:** 2012-03-29

**Authors:** Gustavo Deco, Etienne Hugues

**Affiliations:** 1 Theoretical and Computational Neuroscience Group, Center of Brain and Cognition, Universitat Pompeu Fabra, Barcelona, Spain; 2 Institució Catalana de Recerca i Estudis Avançats (ICREA), Universitat Pompeu Fabra, Barcelona, Spain; University of Oxford, United Kingdom

## Abstract

It is well established that the variability of the neural activity across trials, as measured by the Fano factor, is elevated. This fact poses limits on information encoding by the neural activity. However, a series of recent neurophysiological experiments have changed this traditional view. Single cell recordings across a variety of species, brain areas, brain states and stimulus conditions demonstrate a remarkable reduction of the neural variability when an external stimulation is applied and when attention is allocated towards a stimulus within a neuron's receptive field, suggesting an enhancement of information encoding. Using an heterogeneously connected neural network model whose dynamics exhibits multiple attractors, we demonstrate here how this variability reduction can arise from a network effect. In the spontaneous state, we show that the high degree of neural variability is mainly due to fluctuation-driven excursions from attractor to attractor. This occurs when, in the parameter space, the network working point is around the bifurcation allowing multistable attractors. The application of an external excitatory drive by stimulation or attention stabilizes one specific attractor, eliminating in this way the transitions between the different attractors and resulting in a net decrease in neural variability over trials. Importantly, non-responsive neurons also exhibit a reduction of variability. Finally, this reduced variability is found to arise from an increased regularity of the neural spike trains. In conclusion, these results suggest that the variability reduction under stimulation and attention is a property of neural circuits.

## Author Summary

To understand how neurons encode information, neuroscientists record their firing activity while the animal executes a given task for many trials. Surprisingly, it has been found that the neural response is highly variable, which a priori limits the encoding of information by these neurons. However, recent experiments have shown that this variability is reduced when the animal receives a stimulus or attends to a particular one, suggesting an enhancement of information encoding. It is known that a cause of neural variability resides in the fact that individual neurons receive an input which fluctuates around their firing threshold. We demonstrate here that all the experimental results can naturally arise from the dynamics of a neural network. Using a realistic model, we show that the neural variability during spontaneous activity is particularly high because input noise induces large fluctuations between multiple –but unstable- network states. With stimulation or attention, one particular network state is stabilized and fluctuations decrease, leading to a neural variability reduction. In conclusion, our results suggest that the observed variability reduction is a property of the neural circuits of the brain.

## Introduction

Traditionally, neuroscience aims to discover the neural mechanisms underlying perceptual, cognitive and motor functions by examining neural responses as subjects repeatedly perform a behavioral task. Typically, neural responses are extracted by averaging over those trials and the obtained firing rates are often the only information retained. This approach discards the high firing irregularity and the high variability across trials that individual neurons activity exhibit [1], [2], fluctuations that a priori limit information encoding. At different scales, high fluctuations are also observed in the so-called ongoing activity, and have been shown to play a role on the task-induced activity [3]–[6]. Therefore, the challenging question is: on a single-trial basis, how and in which conditions these a priori detrimental fluctuations allow an efficient information encoding?

Recent experimental studies have examined the neural variability across a variety of species, cortical areas, brain states and stimulus conditions [7]–[10]. Measuring the neural variability with the Fano factor, the mean-normalized variance of the neural spike counts over trials, these studies have found that stimuli generally reduced neural variability [10], in line with previous results in the visual system [11]. Additionally, neural variability has been found to decrease in an attentional paradigm [7], [9].

Theoretically, using a rate model, a recent study [12], [13] has proposed that variability reduction arises from a stimulus induced suppression of an otherwise chaotic ongoing state. Using a spiking network model, we demonstrate here that the variability reduction can arise from an alternative network effect presented in the framework of attractor networks. The formalism of attractor dynamics offers a unifying principle for representation and processing of information [14]–[18]. Co-activation of neurons induces stronger mutual synaptic connections, leading to assembly formation. Reverberatory activity between assembly members can then lead to memory by the persistence of neural activation. The concept of neural assemblies was later formalized in the framework of statistical physics [14]–[16], where these co-activated neurons lead to attractors in the phase space of the recurrent neural dynamics: patterns of co-activation can represent fixed points from which the dynamical system evolves. In this framework, we show that during spontaneous activity, as measured by the mean-normalized variance of the spike count (the Fano factor), neural variability is high when the network exhibits noise-driven excursions between multiple attractors. The application of an external stimulation stabilizes one specific attractor and suppresses the excursions between different attractors, leading to a reduction of neural variability. After an exhaustive study of the Fano factor changes in the network, we conclude that variability reduction is associated with one fundamental condition, namely that in the spontaneous condition, the network working point is around the edge of the bifurcation above which multiple stable (multistable) activated attractors appear. Moreover, we show that the reduced variability can be attributed to an increased regularity of the spike trains, as measured by the coefficient of variation (CV) of the interspike interval (ISI) distribution.

## Results

We study the reduction of neural variability using a spiking neural network model (see Materials and Methods). Real biological networks have a priori an heterogeneous connectivity structure, particularly due to synaptic plasticity and learning: the present network model is an example of this. We first investigate the neural activity and its variability when the network is in a spontaneous state. Then, we consider the effect of the application of an external stimulation to one neural population and how variability is reduced relative to the spontaneous state. Therefore, we study how attention reduces the variability, when the top-down attentional bias is modeled as an increase in the external stimulation. We compare these results with experimental measurements [10], [7], [9]. As a model prediction, we consider the case where two stimuli are presented to different network populations -mimicking the case of two simultaneously presented stimuli in a neuron's receptive field, and when attention is directed towards one of them. We analyze under which conditions the variability is maximally reduced, i.e. when the network best encodes an external stimulation, and conclude that it occurs when the network working point in the spontaneous state is around the bifurcation where multiple stable attractor states emerge. The application of an external excitatory drive (sensory or attentional input) stabilizes one specific attractor, eliminating in this way the transitions between different attractors, resulting in a net decrease in neural variability.

### Stimulation Effects on Variability

**Figure 1:**
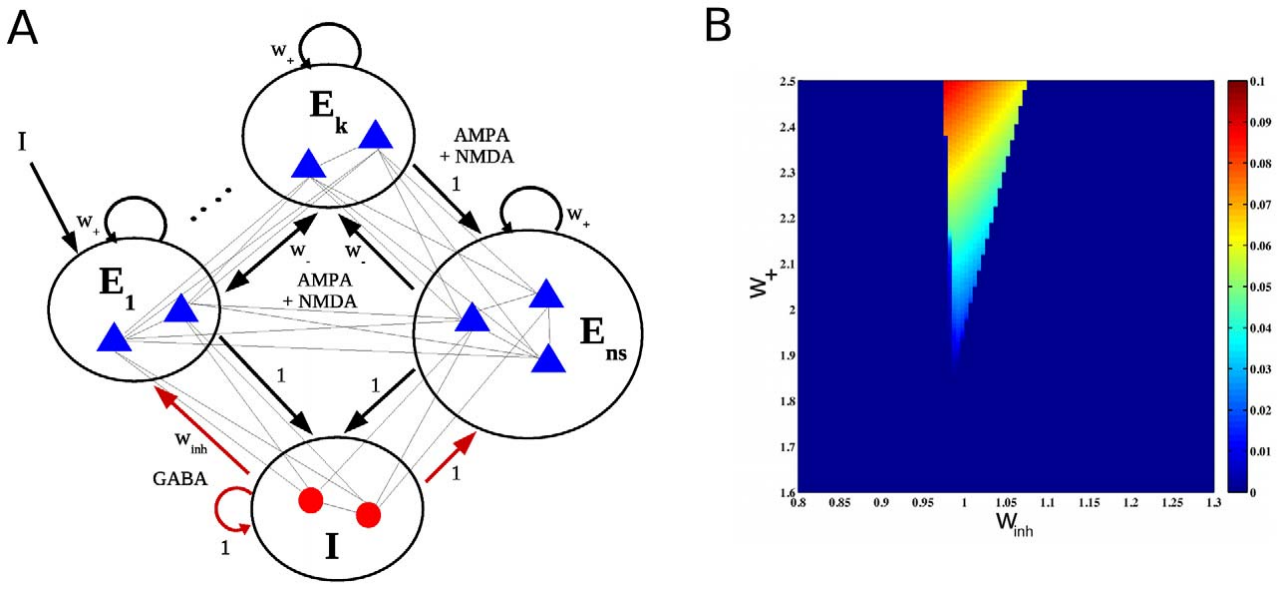
Network architecture and its dynamical behavior to analyze the stimulation and attention effects. (**A**) Network architecture. The network contains excitatory pyramidal cells (blue triangles) and inhibitory interneurons (red circles). Neurons are fully connected and clustered into excitatory and inhibitory (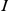) populations (large circles). There are two subtypes of excitatory populations, namely selective (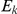) and non-selective (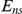), whereby selective populations encode specific stimuli. Edges indicate recurrent connections between neurons in a population, and arrows indicate the connections strengths (see Materials and methods). (**B**) Bifurcation diagram of the network obtained via the mean-field approximation. The rate difference (in kHz) between attractors is plotted, revealing the multistability region (colors except dark blue) where two different types of stable attractors coexist (see Results).

In the attractor network considered here (see Figure 1A and Materials and Methods), each excitatory population is selective for a specific external stimulus. In each of these populations, the recurrent weights are therefore assumed to have increased by Hebbian learning mechanisms to a value 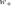, called the cohesion level. The inhibition level 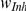 is regulated by the GABA synaptic connections provided by the inhibitory population. The stationary and stable states (attractors) of this network can be studied using a standard mean-field approximation [16], [19], applied here to the case when input rate fluctuations are absent. Figure 1B plots the obtained bifurcation diagram in the spontaneous condition. The diagram shows the firing rate difference between the possible stationary states as a function of the cohesion and inhibition levels. Two regions can be distinguished. First, a large region where there is a unique stationary stable state (large dark blue region): this state corresponds to a low activation state where excitatory and inhibitory neurons fire at a low mean rate (approximately 3 Hz and 9 Hz when 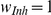, respectively). Second, above a bifurcation, in a region of intermediate inhibition level and sufficiently large cohesion level, the network exhibits multistability with the coexistence of 6 stable states, namely: 5 equivalent states where only one of the 5 specific populations is highly activated, and a low activation state as described above. This last attractor remains stable just above the bifurcation, because of a so-called subcritical bifurcation. Below the bifurcation, although the activated attractors are unstable, fluctuations can induce transient excursions of the network state towards them, the dynamics around these attractors being partially stable even if not globally.

**Figure 2:**
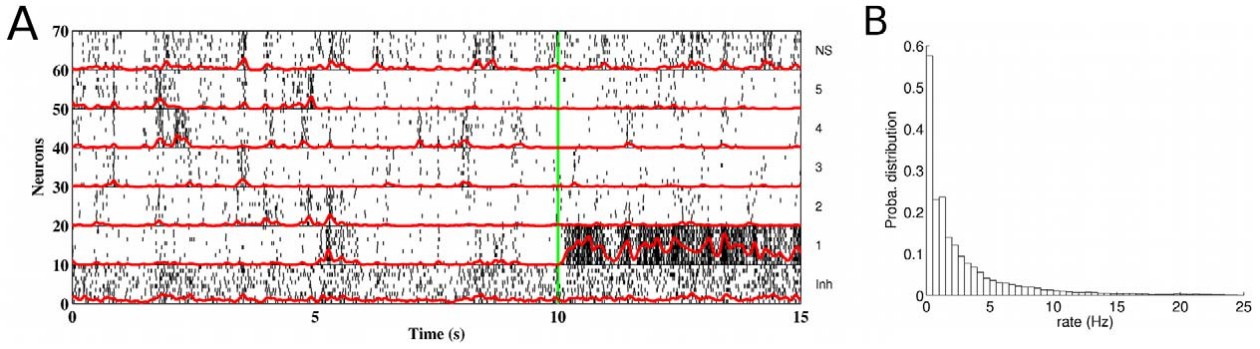
Network firing activity during spontaneous and stimulated conditions. The network cohesion and inhibition levels are 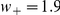 and 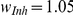, respectively. (**A**) Firing activity for network neurons (10 neurons in each population, as indicated on the right): raster plot (black) together with the mean rate (red curves) for each population, obtained as the convolution of the spike trains with a unit Gaussian function with 50 ms width. For the first 10 s, no stimulus is applied and the resulting ongoing activity is similar in all excitatory populations: in each of them, the firing rate fluctuates irregularly and abruptly as the result of input rate fluctuations and induced excursions between the different attractors of the network. Besides an attractor corresponding to low activation in all populations, there are attractors corresponding to higher activation in one selective population and low activation in all the others. From time 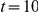 s (green vertical line), a stimulus is presented to the selective excitatory population 1 (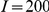 Hz), increasing its mean rate and decreasing the one of the other selective pools. Moreover, all rates have now lower fluctuations: the attractor corresponding to a high activation of population 1 and a low activation of the others is stabilized. (**B**) Probability distribution of the rate fluctuations for the specific excitatory populations during spontaneous activity. The rate fluctuations are quite large (mean rate: 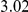 Hz; standard deviation: 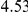 Hz), with no peak except at zero rate and a long tail. This behavior reveals the excursion regime between the different attractors.

First, we focus on spontaneous activity. For a network working point around but below the bifurcation, for example for a cohesion level 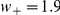 and an inhibition level 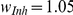, Figure 2A show the network spontaneous activity (for times less than 10 s). The activity is irregular, not only from the timing of spikes but at the rate level, where abrupt changes occur from time to time. Each neural population has different rate fluctuations. The rate distribution for all selective populations (see Figure 2B) is large (mean rate: 3.02 Hz; standard deviation: 4.53 Hz), with a unique peak at zero rate and with a long tail. These rate fluctuations cannot be explained solely by the input rate fluctuations: they reveal the excursions of the activity between the different network attractors. This type of network dynamics may be at the origin of the similar large firing rate fluctuations observed in the cortex of behaving monkeys (Reynolds and Mitchell, personal communication). When an external stimulus is applied to a given population (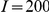 Hz to population 1 at time 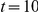 s in Figure 2A), not only the activity of this population increases but the neural activity fluctuations due to the wanderings between the different attractors is sharply reduced. Actually, under stimulation, only the attractor corresponding to high activity in this population and low activity in all others is stable.

**Figure 3:**
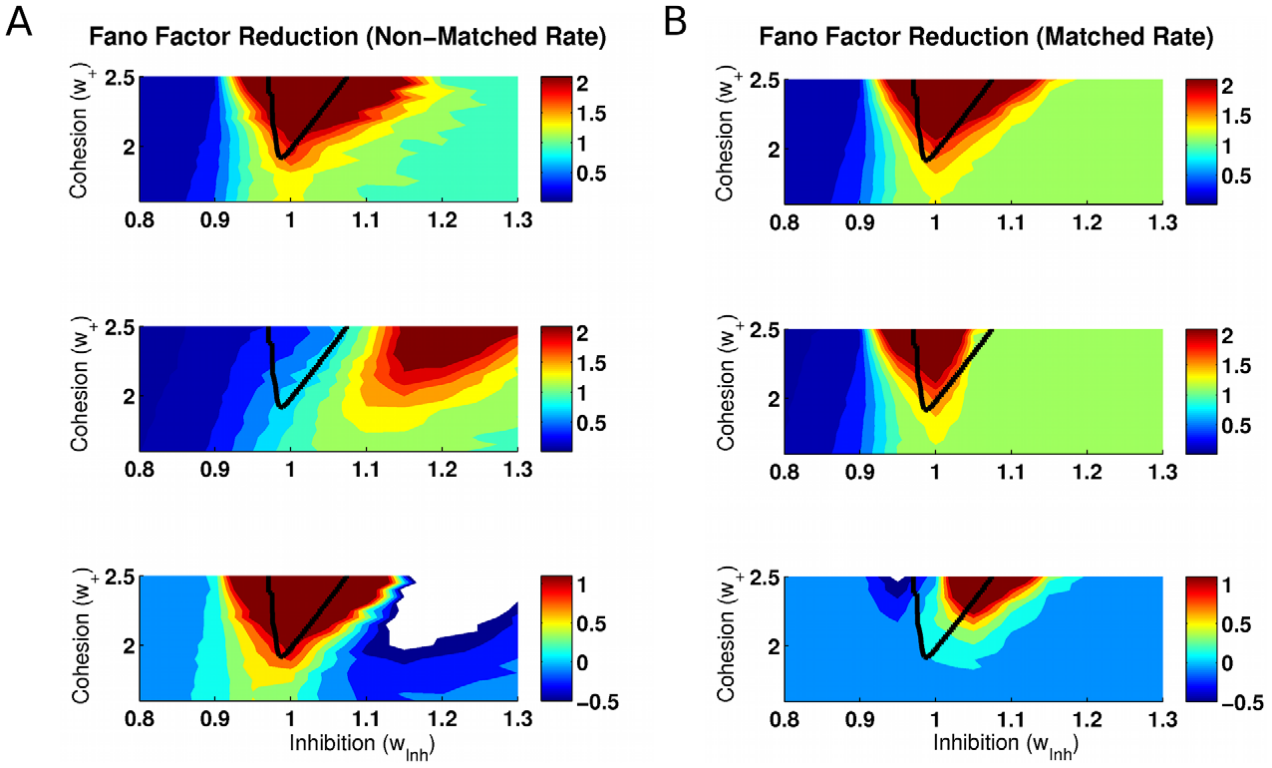
Fano factor as a function of the cohesion and inhibition levels in the (A) non-matched rate and (B) matched rate conditions. Fano factor (top) without and (middle) with external stimulation (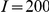 Hz) and (bottom) their difference, i.e. the stimulus driven reduction of the Fano factor. The difference in Fano factor between two consecutive contours is 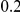. For the Fano factor reduction, the interval 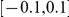 is indicated by the light blue color, and values less than 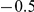 are indicated in white. These results show that a reduction of the Fano factor consistent with the experiments occurs indeed around the bifurcation line, for sufficient inhibition (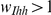).

To study the changes in the neural variability in the network when a specific external stimulus is applied, we investigate the Fano factor reduction corresponding to the difference between the spontaneous condition and when one selective excitatory population is externally stimulated by a Poisson spike train with rate I. The Fano factor was calculated from scatter plots of the neural spike count variance versus mean by linear regression fits constrained to pass through the origin. The spike counts variance and mean were calculated for each neuron separated in windows of 100 ms and averaging over 1000 trials. Applying the mean-matched procedure of Churchland et al. [10], neurons in the stimulated population define the non-matched case (as stimulation increases their rate), whereas neurons in the non-stimulated populations define the matched case (as stimulation changes only slightly their rates). Figure 3 plots the Fano factor without external stimulation, with external stimulation (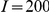 Hz) and their difference as a function of the cohesion and inhibition levels. Figure 3A and 3B show the results for the matched condition and for the non-matched condition, respectively. Both cases show that a reduction of the Fano factor consistent with the experimental findings occurs around the bifurcation line (black line). More precisely, the matched rate case shows that multistability is required for the Fano factor to change. The Fano factor is reduced on the right part of the bifurcation line, for 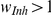, requiring then sufficient inhibition. For the non-matched rate case, the Fano factor is well reduced around the whole multistability region. Note that, due to the presence of noise, multiple attractors manifest themselves outside the region calculated with the mean-field approach (see Figure 1B). Furthermore, the Fano factor level for those regions is consistent with the observed experimental values, namely: around 1.4 for the non-stimulated case and 1 for the stimulated case.

**Figure 4:**
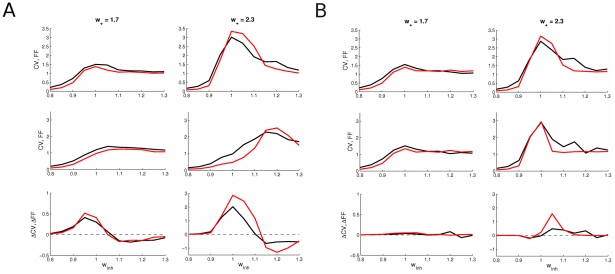
Comparison of the 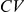
 **(black) and the Fano factor (red) in the (A) non-matched rate and the (B) matched rate conditions for two different cohesion levels.** 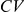 and Fano factor (top) without and (middle) with external stimulation (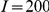 Hz) and (bottom) their difference, i.e. their stimulus driven reduction. The 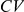 and Fano factor exhibit very similar values and behavior with respect to parameter changes. For a low cohesion level (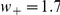, left column), the reduction is lower, and even non-existent in the matched rate case, than for a larger cohesion level (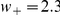, right column) for which the multistability region exists.

Beyond the changes observed over trials, the spiking statistics, like the neural ISI distribution, is also likely to change under stimulation, although it has not been reported experimentally. To compute the ISI distribution, we have simulated the network activity over long time intervals (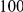 s), and have characterized the ISI distribution by its coefficient of variation (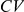). We have compared the 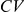 and the Fano factor for two values of the cohesion level 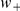, a low value for which there is no multistability (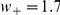) and a high value for which there is (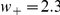). As shown in Figure 4, the CV and the Fano factor are always very similar, and vary similarly across all conditions. Therefore, our model results suggest that the Fano factor change is due to an underlying change in the spiking statistics.

**Figure 5:**
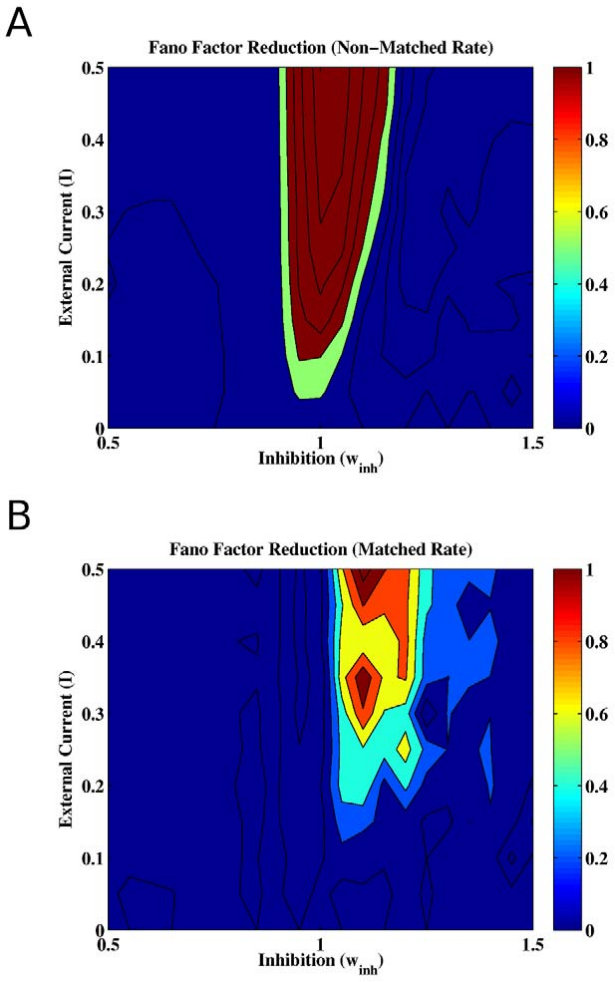
Fano factor reduction for a fixed cohesion level ( 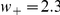
 **here) as a function of inhibition level and stimulation strength.** In the stimulated condition, one selective excitatory population is externally stimulated at a rate 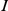 (in kHz). (**A**) The non-matched condition corresponds to the 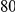 neurons of the stimulated population and (**B**) the matched-condition to the 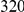 neurons belonging to the other non-stimulated populations, using the mean-matched procedure of Churchland et al. (2010).

For a fixed cohesion level allowing multistable states, Figure 5 shows for the non-matched and matched conditions the Fano factor reduction as a function of the external stimulation and inhibition levels. For the stimulated neurons, the neural variability reduction appears in regions where the spontaneous state is around the bifurcation and increases with the external stimulation. For the non-stimulated neurons, the reduction of neural variability emerges also in the same region. In the spontaneous state, neural variability is high because of the network state excursions between different attractors. The application of an external stimulation stabilizes one specific attractor and suppresses the wanderings between different attractors (see Figure 2A), leading to a neural variability reduction.

**Figure 6:**
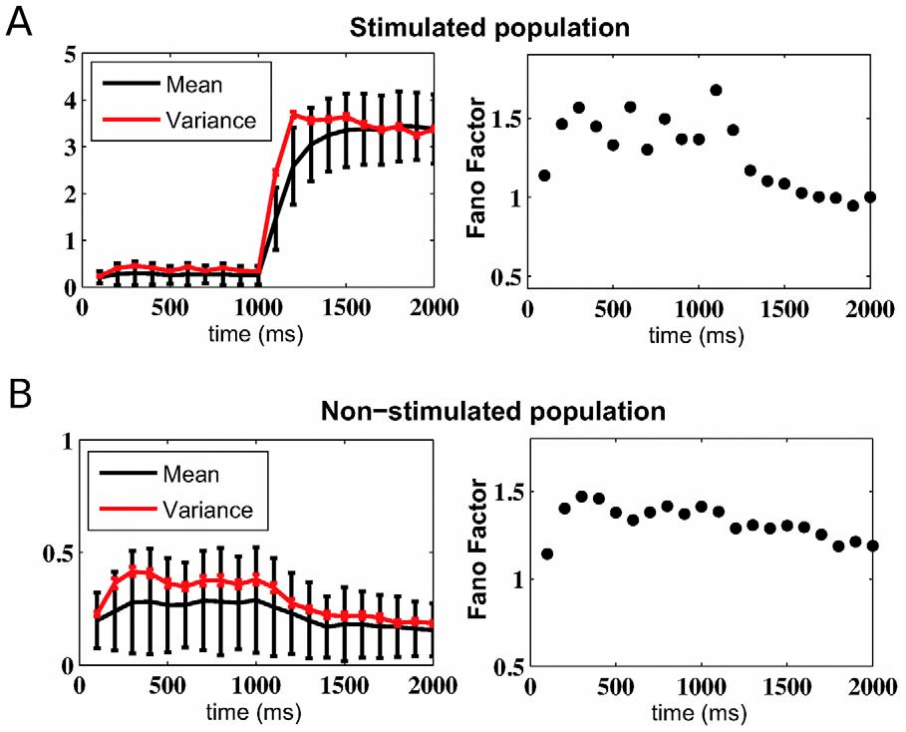
Changes in the spike count mean, variance and Fano factor due to the stimulation of one specific population. The network cohesion and inhibition levels are 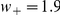 and 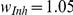, respectively. After a first period of 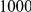 ms without stimulation, a specific external stimulation was applied during 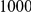 ms onto the neurons in the selective population 1. (**A**) Averaged results obtained with the 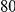 neurons of the stimulated population and (**B**) with the 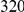 neurons of the other non-stimulated populations.

Figure 6 shows in more detail the evolution of the spike count mean, variance and Fano factor as a function of time for a network with a cohesion level 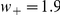 and an inhibition level 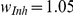, as in Figure 2. The first 1000 ms were simulated in the spontaneous condition. A specific external stimulation was applied from 1000 to 2000 ms to the selective neural population 1. The top part of Figure 6 corresponds to the averaged results obtained from the 80 neurons of the stimulated population 1, whereas the bottom part corresponds to the averaged results from the 320 neurons in the other non-stimulated populations. For the stimulated neurons, the spike count mean and variance increase when the stimulus is applied (at 1000 ms), but in such a way that the Fano factor is reduced. For the non-stimulated neurons, the stimulation effect is to reduce both spike count mean and variance, in a way that reduces the Fano factor. The Fano factor was calculated from the spike count variance versus mean scatter plots, where each point represents one neuron, the Fano factor being deduced using a linear regression fit (see Materials and methods).

### Attentional Effects

**Figure 7:**
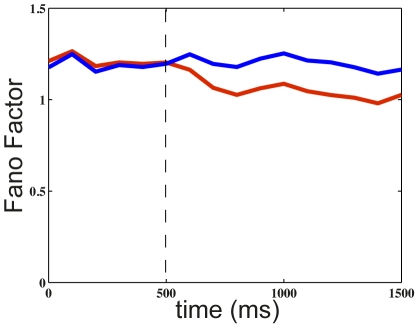
Fano factor without and with attention in the case of one stimulus. After 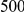 ms of stimulus presentation to a given selective population (with rate 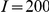 Hz), attention is allocated (with bias 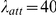 Hz) (red curve). Comparison to the case without attention (blue curve).

Recent experiments have studied the effect of attention on the neural variability over trials [7], [9], Single V4 cells were recorded in awake behaving monkeys when one stimulus in the neuron's receptive field was behaviorally attended or not. Both studies reported a relatively small but significant decrease of the Fano factor in the attended condition with respect to the non-attended one. In our simulations, we modeled the attentional bias by increasing the level of exogenous input to the stimulus-specific population when this stimulus was attended, similar to an increase of the stimulus-related input. In this sense, it can be regarded as a baseline shift mechanism, or an increase in contrast (However, it does not accommodate any attentional gain mechanism that would be better modeled through changes in postsynaptic sensitivity, possibly through NMDA receptor dynamics). As the neural variability reduction increases with stimulus strength (see Figure 5), the application of an attentional bias therefore decreases the Fano factor compared to the non-attended case. The effect of attention on the Fano factor evolution in shown in Figure 7 by comparing with the non-attended case. These results show that the model reproduces the range of Fano factor reductions observed experimentally [7], [9].

**Figure 8:**
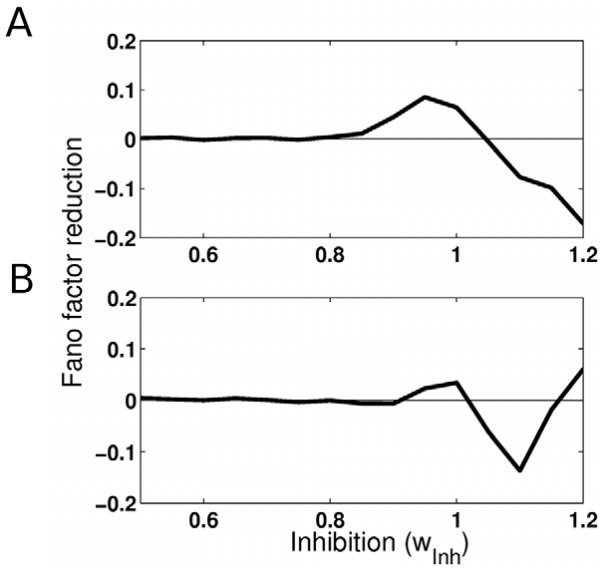
Fano factor reduction as a function of the inhibition level in the biased competition case for cohesion level 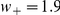
 **.** Two selective populations receive a stimulus, and attention is allocated to one of them. The Fano factor is averaged over the 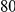 neurons in each stimulated population. Fano factor reduction (**A**) for the attended stimulus corresponding population and (**B**) for the non-attended one.

As a model prediction, we consider now the case where two stimuli are presented simultaneously in a neuron's receptive field and when attention is allocated to only one of them, a situation referred to lead to “biased competition” [20], [21]. In the network, selective populations 1 and 2 encode each one of the two simultaneously presented visual stimuli: the target which should be attended and the distractor which should be ignored. After 500 ms of spontaneous activity, the two external visual stimuli are applied to these two populations for 1000 ms. In the attended condition, the selective population corresponding to the target receives the attentional bias. Figure 8 plots the neural variability reduction for the cohesion level 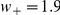. The variability reduction is obtained as the difference of the average Fano factor for the 80 neurons in the attended (Figure 8A) versus in the non-attended stimulated populations (Figure 8B). For both populations, variability is reduced by attention around the region where the system is multistable without attention (around 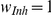). The mechanism responsible for the reduction of neural variability is identical to the one described above. In the condition without attention noise-driven excursions between attractors generates a high neural variability. Allocation of attention stabilizes one of the attractors, namely the one corresponding to higher activation of the attended target population and lower activation of the ignored distractor population.

## Discussion

In the spontaneous or undriven state, why do cortical circuits exhibit a relatively high degree of neural variability across trials? Why does this variability decrease when a stimulus is presented or when attention is paid? Here, we investigated what could underlie these phenomena in a realistic neural network. Our results show that, under spontaneous conditions, the high degree of neural variability in a neural circuit could essentially be due to fluctuation-driven excursions between the different attractors of the circuit dynamics. This is possible if, in the parameter space, the spontaneous state of the circuit resides around the edge of a bifurcation above which multistable attractors appear. The application of an external excitatory drive, either mediated by a sensory stimulus or by attention, stabilizes one specific attractor and suppresses in this way the transitions between the different attractors. This results in a net decrease in neural variability as measured as a by the Fano factor. More precisely, the matched rate case shows that multistability is required for the Fano factor to change. The Fano factor is reduced on the right part of the bifurcation line, requiring then sufficient inhibition (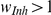). For the non-matched rate case, the Fano factor is well reduced around the whole multistability region. In conclusion our results show that, in the model parameter space, there exists a region where the Fano factor is reduced, both in the non-matched and the matched rate case. Because the spike count signal-to-noise ratio is increased, this reduction suggests an improved encoding of the external signal.

Beyond the variability over trials, we have also shown that the 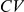 of the neural ISI distributions varies similarly to the Fano factor across all conditions, meaning that the variability reduction is due to a concomitant increase of the spike trains regularity. It would be interesting to verify this model prediction experimentally. However, due to relatively short recorded time intervals, this quantity may be difficult to measure and the 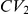 [22], which requires only the knowledge of two consecutive ISIs, could be employed instead.

The above conclusions have been obtained for the present heterogeneously connected network and rely on the existence of multiple attractors. However, the present scenario does not depend on the specific network structure, provided the network exhibits multistability. In this case, there is a region of the parameter space where there is strict multistability, meaning the co-existence of multiple stable states. From dynamical systems theory, it is known that close to this region, the unstable attractors can still transiently attract the dynamics, a behavior which will be favored by fluctuations. Note that, because multistability is needed here to reproduce the experimental observations, this excludes a priori single neuron mechanisms.

In a recent study [12], [13], the authors have proposed that the ongoing spontaneous activity is chaotic, and that stimulation suppresses this chaos. They use a phenomenological firing rate model which allows a theoretical understanding of the network behavior. The realism of the present model allows a quantitative comparison with experimental results but prevents at the same time such a theoretical understanding. Only could we predict the stationary states of the network using a mean-field approach. Beyond the naïve analogy that stimulation suppresses ongoing fluctuations in the two models, a number of differences between the two models (in their case: rate model, no noise, temporal input, phase transition) indicate that the two scenarios are different.

Further experimental and theoretical evidence supports the present scenario. At the microscopic level and using optical imaging, Arieli et al. [4] (see also [5]) first showed that spontaneous activity is highly coordinated across large neural assemblies in the primary visual cortex (V1) of an anesthetized cat. Furthermore, the pattern of co-activation is feature-specific in the discharge of individual neurons and is temporally locked to the activation of other cells with similar orientation preferences whose spatial organization is described by orientation maps. Finally, the variability of such ongoing activity can explain much of the variability in subsequent sensory-evoked responses, indicating a potential link with perception. Blumenfeld et al. [23] accounted for this type of cellular ongoing activity by assuming that this activity resulted from noise-driven transitions between multistable attractors of the intracortical network. They suggested a rate model endowed with a simple local connectivity rule, and showed that it yields attractor states that are highly similar to the orientation maps alternatively activated in the absence of stimulation. They also considered the case where the activity is evoked by a visual stimulus and showed how a structured afferent input can select the orientation map that matches the orientation of the stimulus. Their model therefore suggests that orientation maps are encoded in the lateral connections, and that these connections can generate an orientation map both when the activity is spontaneous and when it is evoked by a visual stimulus.

In a recent work, where the same biophysical spiking neural network was used, it was shown that, using the Fisher information, the network neural activity best encoded a small external input, or modulatory input (like it is believed to be the case for attention), in the region of the parameter space where excitatory and inhibitory input currents almost balance each other, and which correspond to 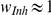 (Deco and Hugues, PLoS One, in press). This regime of balanced input is supported by experimental evidence in vitro [24] and in vivo [25], [26]. Taken together with the present results, we can conclude that, in the region of the parameter space around the bifurcation and where input currents almost balance, our model agrees with all the experimental findings, which suggests that these observations may also correspond to a region of best stimulus encoding.

At the global level of large-scale neural systems, a broad body of experimental work, mainly using the BOLD fMRI hemodynamic response, has suggested that brain activity during resting state is not random but has a spatiotemporal structure (for a review see [27]). From these findings, ongoing neural activity may therefore be organized in a series of functional networks, so-called resting state networks. Suggested by a modeling study, these networks may also emerge from noise-induced transitions between multiple oscillatory brain states [28]. In this sense, the model of Blumenfeld et al. [23] at the microscopic cellular level, and the model of Deco et al. [28] at the global neuroanatomical level, propose that spontaneous ongoing activity is built up with multiple attractors, each one related with different specific stimulations or tasks, and that this activity fluctuates during spontaneous activity (or rest) due to transitions between those attractor states, induced by noise and unstructured input.

## Materials and Methods

To study the variability changes due to stimulation or attention allocation, we consider a biophysically realistic neural network model, at the same time sufficiently simple to allow a theoretical investigation of its dynamical behavior [16]. We have chosen such a biophysical model to be able to reproduce quantitatively the experimental findings. The model uses integrate-and-fire neurons with excitatory (AMPA and NMDA) and inhibitory (GABA-A) synaptic receptor types. It is formulated and analyzed in the theoretical framework of attractor networks introduced in the seminal work of Amit [14]. An attractor network is a neural network whose dynamical state has the tendency to settle into a stable pattern of firing, which eventually destabilizes under the effect of noise. Its behavior can be formally described by dynamical systems theory.

### Neurons and Synapses

The spiking activity of neurons in the network is described by an integrate-and-fire model. Integrate-and-fire (IF) neurons are point-like elements, whose dynamical state is described by their membrane potential 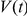. An IF neuron can be described by a basic circuit consisting of a cell membrane capacitance 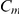 in parallel with a membrane resistance 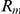, driven by input currents coming from connected neurons. Hence, the subthreshold dynamics of the membrane potential of each neuron in the network is given by the following equation:
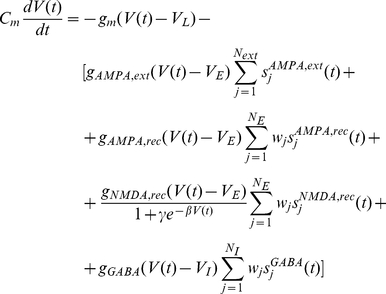
(1)where 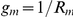 is the membrane leak conductance, 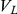 is the resting potential, and 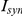 is the synaptic current. The membrane time constant is defined by 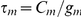. When the voltage across the membrane reaches a given threshold 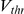, the neuron generates a spike which is then transmitted to other neurons and the membrane potential is instantaneously reset to 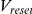 and maintained there for a refractory time 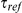 during which the neuron is unable to produce further spikes. The spikes arriving to a given neural synapse produce an input to the neuron which induce post-synaptic excitatory or inhibitory potentials (through a low-pass filter formed by the membrane and synaptic time constants). In Equation 1, 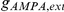, 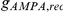, 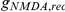, and 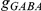 are the synaptic conductances, and 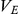, 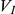 the excitatory and inhibitory reversal potentials, respectively. The dimensionless parameters 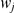 of the connections are the synaptic weights. The NMDA currents are voltage dependent and they are modulated by intracellular magnesium concentration. The gating variables 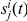 are the fractions of open channels of neurons and they are given by:
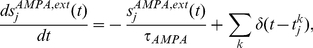
(2)

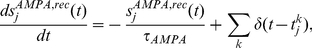
(3)

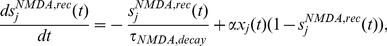
(4)

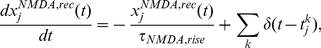
(5)

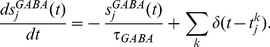
(6)The sums over the index 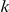 represent all the spikes emitted by the presynaptic neuron j (at times 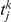). In Equations 2–6, 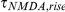 and 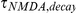 are the rise and decays times for the NMDA synapses, and 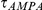 and 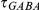 the decay times for AMPA and GABA synapses. The rise times of both AMPA and GABA synaptic currents are neglected because they are short (<1 ms). The values of the constant parameters and default values of the free parameters used in the simulations are displayed in Table 1.

**Table 1:** Neural and synaptic parameters.

Excitatory neurons		Inhibitory neurons		Synapses	
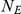	800 neurons	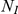	200 neurons	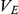	0 mV
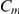	0.5 nF	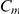	0.2 nF	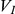	−70 mV
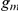	25 nS	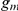	20 nS	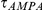	2 ms
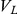	−70 mV	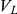	−70 mV	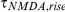	2 ms
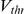	−50 mV	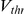	−50 mV	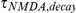	100 ms
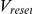	−55 mV	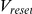	−55 mV	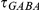	10 ms
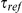	1 ms	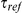	1 ms	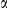	0.5 ms^−1^
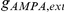	2.08 nS	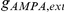	1.62 nS	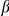	0.062 mV^−1^
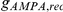	0.104 nS	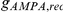	0.081 nS	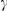	0.2801
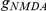	0.327 nS	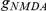	0.258 nS		
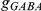	1.25 nS	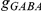	0.973 nS		

This table gives the values used in the numerical simulations for the parameters that enter in the definition of neuron and synaptic models (See Materials and Methods).

### Neural Network

We use the same network to study both situations, namely the effects of stimulation and attention on the neural variability over trials. The network consists of 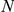 (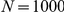 in our simulations) interacting neurons, where 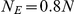 are excitatory (pyramidal) cells and 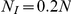 are inhibitory cells (interneurons), consistent with the neurophysiologically observed proportions [29]. We use an attractor network where neurons are organized into a discrete set of populations (see Figure 1A). There are three different population types, namely: 1) the inhibitory population, 2) the excitatory non-selective population and 3) the excitatory selective population. The inhibitory population 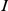 is made of the inhibitory neurons in the modeled brain area and mediates competition in the attractor network by distributing a global inhibitory signal. The non-selective population 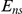 is composed of all excitatory neurons that are not receiving any specific external input and which therefore provides a background level of excitation. The remaining excitatory neurons are clustered in different populations 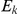, 5 in the simulations reported here. Each contains 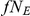 neurons (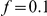 in our simulations) which are sensitive to a specific external stimulus. The network is fully connected, meaning that each neuron in the network receives 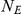 excitatory and 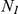 inhibitory synaptic contacts. The connection strengths between and within the populations are determined by dimensionless weights 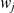. We assume that the connections are already formed, e.g. by earlier self-organization mechanisms, as if they were established by Hebbian learning, with the coupling between two neurons being strong if their activities are correlated and weak if they are anticorrelated. The recurrent self-excitation within each selective population 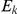 is given by the weight 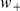 (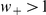), which is called the cohesion level, and the weaker connection between them by the weight 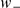 (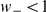). The synaptic efficacy 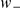 depends on 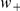 by the relation 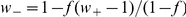. This serves to ensure that the average excitatory synaptic efficacy will remain constant as 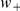 is varied across conditions. Neurons in the inhibitory population are mutually connected with an intermediate weight 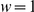. These neurons are also connected with all excitatory neurons with the same intermediate weight, which for excitatory-to-inhibitory connections is 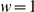 and, for inhibitory-to-excitatory connections, is denoted 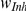 and called the inhibition level. Neurons in each excitatory population 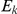 are connected to neurons in the population 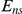 with a feedforward and feedback synaptic weights 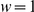 and 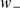, respectively. The remaining connections are all set to the baseline value, i.e. to 1.

All neurons in the network always receive an external background input from 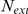 external neurons emitting uncorrelated Poisson spike trains at rate 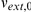. The resulting spike train is still a Poisson spike train, with rate 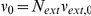. More specifically, and for all neurons inside a given population p, the resulting spike train is assumed to have a time-varying rate 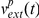, governed by
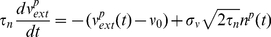
(12)where 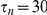 ms, 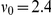 kHz, 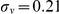 kHz is the standard deviation of 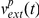 and 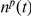 is a normalized Gaussian white noise. Due to noise, negative values of 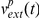 that could arise are rectified to zero. These input rate fluctuations represent the noisy fluctuations that are typically observed *in vivo*. Additionally, neurons in a specific selective population 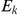 could receive other inputs when an external stimulus is applied or when attention is allocated to that population. These inputs are specified by adding a corresponding rate to the rate of the background Poissonian input spike train.

### Stimulation Effect

Without stimulation, all neurons only receive the background input. In the stimulation case, the first selective population is stimulated, receiving an extra input whose rate is 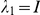. Without external stimulation, the spontaneous activity of the network consists in a noise-driven wandering between different attractors (See Results for more detailed explanations), each corresponding to higher activation in one selective population and lower activation in all the others. Stimulation onset stabilizes one attractor, corresponding to the high activation of the stimulated selective population. The spiking activity for one trial is simulated for 500 ms without stimulation, allowing the network activity to stabilize, and the stimulus is then presented during 100 ms. Results during the stimulus period are averaged over 1000 trials initialized with different random seeds.

### Attentional Effect

We analyze the effect of attention on the neural variability and study the encoding of an attended stimulus. For this, we first address the recent experimental results when one stimulus is presented in a neuron's receptive field [7], [9]. In a second part, as a prediction, we analyze the case where two stimuli are presented in a neuron's receptive field, a case used to elicit “biased competition” [20], [21]. In the model, when attention is applied to a given stimulus, a biasing input corresponding to a rate 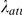 is added to the input of the corresponding stimulus specific population [30], [31]. In the case of one stimulus, the effect of attention is consequently assimilable to an increase of the stimulus strength or contrast, and is essentially a particular case of the stimulation case. In the case of two stimuli, each simulation started with a period of 500 ms (for network activity stabilization). Then, during a period of 1000 ms, an identical stimulus was presented to selective populations 1 and 2, represented by the corresponding extra rates 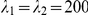 Hz, respectively. Two cases were compared: with and without attention. In the case with attention, an extra attentional bias 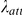 was added to the population 1, corresponding to the attended spatial location (i.e. 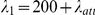 Hz). In the case without attention, no bias was applied. The spiking activity was averaged over 2000 trials initialized with different random seeds.

### Fano Factor

In the spiking simulations, we characterized neural variability using the mean-normalized variance of the spike counts, i.e. the Fano factor. It is defined as 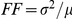, where 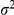 is the variance and 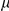 is the mean of the spike counts of a neuron in a time window W. In all cases, we used a time window of 100 ms. The Fano factor measures the noise-to-signal ratio and therefore characterizes the neural variability over trials. For example, for a Poisson process, the variance equals the mean spike count (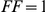) for any length of the time window. We calculated the Fano factor by fitting a linear regression (constrained to pass through the origin) to scatter plots of the spike-count variance versus mean for each of the 80 neurons in the analyzed population. The variance and mean of the spike counts were calculated for each individual neuron by averaging over 1000 trials. In conditions for which the rate of some populations was not significantly affected, which is the case in practice for the non-stimulated neuronal populations, we used the mean-matching procedure for the Fano factor described in Churchland et al. [10] whose aim is to have the same distribution of mean firing rates and therefore factor out the rate for the Fano factor changes. In brief, the mean matching procedure consists of selecting neurons such that the distribution of the spike count for each condition (with or without stimulation) is the same. We took the greatest common distribution of the computed spike count for each condition. Individual points are then randomly removed until the actual distribution matched the common distribution. The mean-matched Fano factor is based on the remaining points.
